# The impact of cancer diagnosis on functional decline in adults aged 50 and older: the US Health and Retirement Study

**DOI:** 10.1007/s11764-025-01867-5

**Published:** 2025-07-29

**Authors:** Gina E. Nam, Elizabeth Rose Mayeda, Yancen Pan, Eleanor Hayes-Larson, L. Paloma Rojas-Saunero, Hua Zhou, Jian Yu Rao, Zuo-Feng Zhang

**Affiliations:** 1 Department of Epidemiology, UCLA Fielding School of Public Health, 71-225 CHS, 650 Charles E. Young Drive South, Box 951772, Los Angeles, CA 90095-1772 USA; 2 Leonard Davis School of Gerontology, University of Southern California, Los Angeles, CA USA; 3 Department of Biostatistics, UCLA Fielding School of Public Health, Los Angeles, CA USA; 4 Department of Computational Medicine, UCLA David Geffen School of Medicine, Los Angeles, CA USA; 5 Department of Pathology and Laboratory Medicine, UCLA David Geffen School of Medicine, Los Angeles, CA USA; 6 UCLA Jonsson Comprehensive Cancer Center, Los Angeles, CA USA; 7 Department of Medicine, Center for Human Nutrition, UCLA David Geffen School of Medicine, Los Angeles, CA USA

**Keywords:** Functional decline, ADL, IADL, Cancer, Older cancer survivors, Older adults

## Abstract

**Purpose:**

We conducted a longitudinal secondary data analysis to estimate long-term functional limitation trajectories of adults over 50 with cancer compared to cancer-free individuals.

**Methods:**

Using the Health and Retirement Study (1998–2020), we followed adults over 50 without cancer history. Incident cancer was self-reported or proxy reported. Functional decline was assessed using self-reported independence in six domains, including instrumental activities of daily living (IADL), activities of daily living (ADL), mobility, large muscle, gross motor, and fine motor skills. We employed linear probability models with repeated measures to estimate independence probabilities.

**Results:**

Among 15,972 participants (mean [SD] age, 66.1 [9.9] years), 23.1% reported cancer during follow-up. Before diagnosis, individuals with cancer had a 1.3% lower probability of IADL independence compared to cancer-free individuals (95% CI =  − 1.9, − 0.7). After diagnosis, cancer survivors experienced a sharp 6% drop in IADL independence, resulting in a 3.4% lower probability than in those without cancer (− 4.6, − 2.2). The annual rate of IADL decline after diagnosis was slower in cancer survivors compared to those without cancer (− 0.9% vs. − 1.3%). Similar patterns were observed for ADL. The greatest immediate reduction was in the gross motor domain with a decline of 8.6% (− 10.4, − 6.8).

**Conclusions:**

Cancer survivors experienced rapid functional decline at diagnosis, possibly attributed to active treatment. Following diagnosis, cancer survivors had a more gradual loss in functional independence compared to cancer-free individuals.

**Implications for cancer survivors:**

Findings underscore the importance of proactive, tailored interventions to support functional independence in older cancer survivors, particularly around diagnosis.

**Supplementary Information:**

The online version contains supplementary material available at https://doi.org/10.1007/s11764-025-01867-5.

## Introduction

Functional status, an individual’s ability to perform daily activities and maintain independent living, is a significant concern for older adults with cancer. From the moment of diagnosis, cancer survivors face a high burden of difficulty in performing daily tasks (i.e., functional limitations) that can persist long after treatment ends [1–3]. In fact, approximately 70% of cancer survivors in the USA reported experiencing functional limitations in 2018 [4]. These limitations may progress into functional decline, defined as the loss of functional independence over time, and can negatively impact overall quality of life [5, 6]. The loss of functional independence can be especially detrimental for older adult cancer survivors, as quality of life is often valued more than extended survival [7]. Therefore, evaluating the long-term impact of functional limitations is an important in this population to support the preservation of their quality of life [6, 8–11].

Older adult cancer survivors may experience an accelerated aging process as a result of cancer and its treatment, leading to earlier and more rapid functional decline compared to individuals without cancer [1, 12]. However, only a few studies have examined long-term functional trajectories in cancer survivors compared to cancer-free individuals to support the accelerated aging model [13–15]. Notably, research has shown that functional decline prior to diagnosis is similar in individuals with and without cancer [13–15]. After diagnosis, a greater functional decline was observed in the cancer group, with the most significant reductions occurring around the time of diagnosis [13, 14]. However, the difference in the functional decline among people with and without cancer may attenuate over time, with one study indicating a slowdown immediately after diagnosis, while another reports a more gradual decline over several years [13, 15].

While functional assessment plays a crucial role in the care of older adults with cancer, much of the current evidence is derived from short-term observations during the cancer diagnosis period, active treatment, or immediately after treatment completion among older adults with cancer. These studies typically measured functional decline at specific intervals—3, 6, 9, and 12 months—following treatment initiation or completion [1, 2]. The existing research shows that older adults with cancer have greater functional decline shortly after diagnosis compared to their cancer-free counterparts [1, 2]. Moreover, accelerated functional decline is significant in advanced stages and during active treatment, with cancer treatment itself thought to be the main factor contributing to this decline [1]. Chemotherapy, hormonal therapy, and radiotherapy lead to functional decline in older patients undergoing these treatments [16–22].

The objective of this study was to examine the long-term functional limitation trajectories among adults aged 50 and older from 1998 to 2020 using data from the Health and Retirement Study (HRS) participants without a history of cancer in 1998. Although definitions and reporting of functional status differ across organizations and fields [23, 24], they consistently emphasize an individual’s ability to perform activities. Building on the previously introduced definitions, we first compared functional decline trajectories between individuals with and without cancer. Additionally, for the cancer group, we examined differences in functional limitation trajectories before and after cancer diagnosis with functional limitation trajectories among cancer-free counterparts. Through this investigation, we aimed to deepen our understanding of long-term functional decline among cancer survivors compared to those who remain cancer-free. By identifying patterns of functional limitations over time, our study contributes to the development of targeted strategies for monitoring and addressing the functional needs of older adult cancer survivors across the cancer care continuum.

## Methods

### Study design and participants

The HRS is a nationally representative longitudinal study of US adults aged 50 and older (RRID:SCR_008930). Participants were selected using a multi-stage area probability design, and a brief household screening interview was conducted to determine eligibility for enrollment [25, 26]. Additionally, the HRS conducts after-death interviews with proxy respondents (e.g., spouses or other family members) of deceased participants to gather information on various aspects related to the end of life [25], including cancer diagnoses, between their last interview and death.

**Fig. 1:**
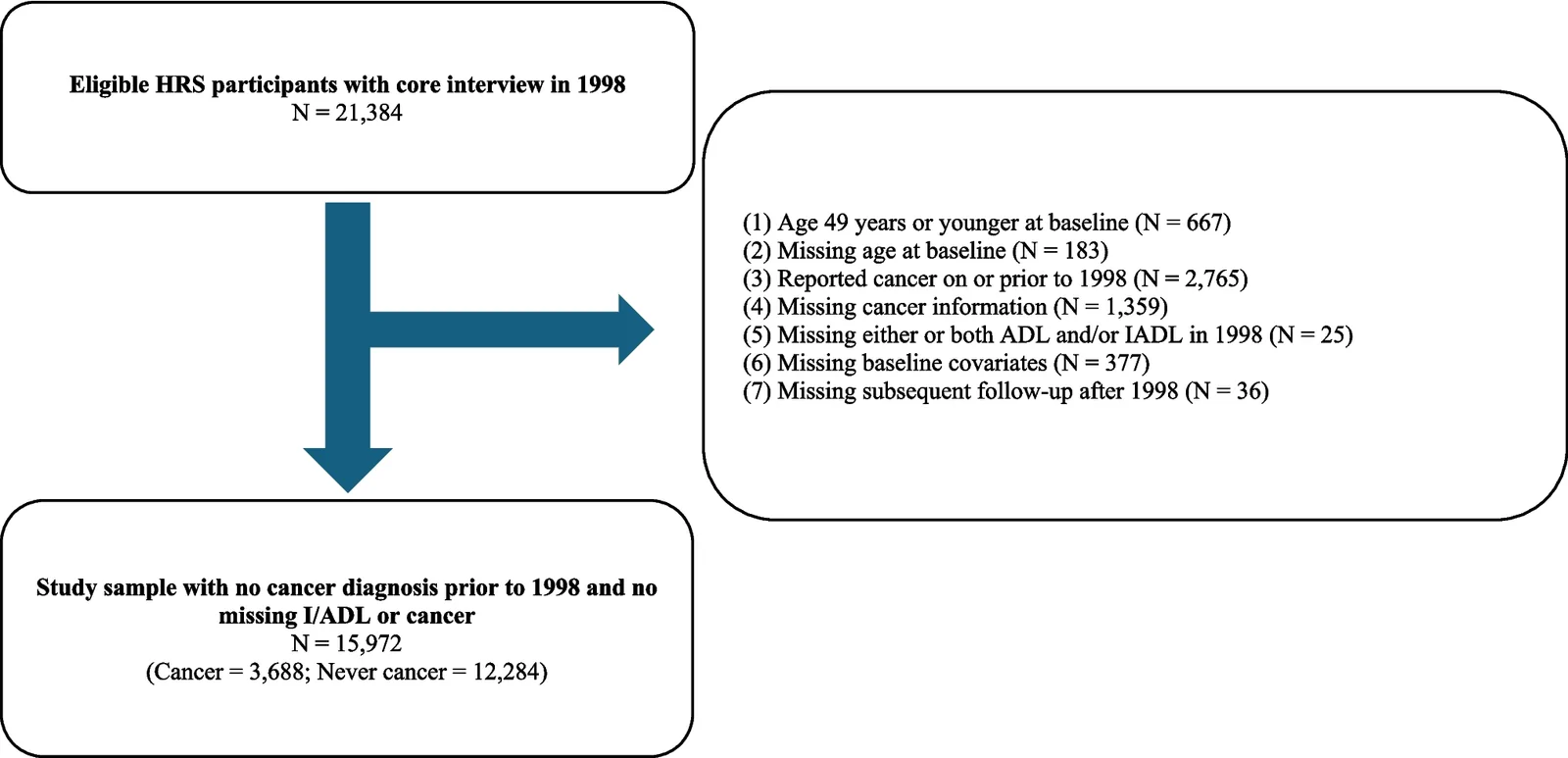
Flowchart of the study sample

This study is a secondary analysis of the HRS data collected biennially from 1998 to 2020. We selected 1998 as the baseline year as opposed to the earliest interview year of 1992, because the survey questions pertinent to our analysis were standardized beginning with the 1998 wave. Therefore, the study sample consisted of 21,384 individuals who completed the 1998 HRS interview. Participant excluded from our study were individuals aged < 50 years at baseline (birth year before 1949; *N* = 677); those who reported a cancer diagnosis in or prior to 1998 (*N* = 2,765); those with missing information about their cancer status throughout the entire study period (*N* = 1,359); those with missing baseline information on interview age (*N* = 183), functional status (*N* = 25), and covariates (*N* = 377); and those with only one measure and no subsequent follow-up (*N* = 36). After applying the exclusion criteria, the final analytical sample included 15,972 participants (Fig. 1).

### Cancer ascertainment

Cancer diagnosis status was biennially collected for each participant with the question, “Has a doctor ever told you that you have *cancer or a malignant tumor, excluding minor skin cancers*?” Participants were given the option to respond with either “yes” or “no.” Cancer newly reported during the follow-up period was considered incident cancer (*n* = 2,578). Participants who reported having cancer were asked to provide additional details about their diagnosis, including the date of diagnosis (year and month), the site of cancer, and the type of treatment they received (i.e., chemotherapy, surgery, radiation, and interventions targeting cancer-related symptoms). To comprehensively capture all participants with a cancer diagnosis, we included incident cancer diagnoses reported through after-death interviews by proxy respondents among deceased participants (*n* = 897); and individuals who had a reported cancer diagnosis date but did not report the occurrence of cancer (*n* = 157). Additionally, for participants without information on date of cancer diagnosis, we used the midpoint between the last interview without a reported cancer diagnosis and the subsequent interview where cancer was first reported (*n* = 56). This approach was done to retain participants with incomplete information regarding their cancer diagnosis date (Supplementary Table S1) [27].

### Assessment of functional status

The HRS included six domains of functioning: instrumental activities of daily living (IADL), activities of daily living (ADL), mobility, large muscle skills, gross motor skills, and fine motor skills [28]. Each measure was self-reported and comprised a set of tasks commonly performed by individuals as a part of their daily routines. These measures have demonstrated strong reliability across HRS cohorts and validity in older adult populations [28]. While proxy interviews were conducted when respondents either declined participation without providing a specific reason or were unable to participate due to physical or cognitive limitations [29], we restricted our analysis to self-reported functional status to ensure consistency in respondents’ perceptions. We defined functional independence as the ability to perform specified tasks within the functional domain without difficulty, whereas functional limitations were defined as difficulty in performing one or more tasks within the functional domain. Accordingly, each outcome was treated as a binary variable, with 1 indicating functional independence and 0 indicating functional limitations. Our co-primary outcomes were IADL independence, consisting of five tasks (using a telephone, taking medication, handling money, shopping, and preparing meals), and ADL independence, consisting of five tasks (bathing, eating, dressing, walking across a room, and getting in or out of bed) [5, 28]. Secondary outcomes included independences in mobility, large muscle skills, gross motor skills, and fine motor skills [5, 28]. A description of tasks used to measure each outcome can be found in the supplementary material (Supplementary Table S2).

### Covariate assessment

We adjusted for demographic and socioeconomic factors, including gender (male vs. female), race (White, Black, or others), educational attainment (less than or equal to high school vs. greater than high school), marital status (married, never married, divorced/separated, or widowed), and total household wealth (per $1,000) [1, 14, 30, 31]. We included additional covariates to account for potential confounding between cancer and functional decline: tobacco use (ever smoking vs. never smoking), alcohol use (ever alcohol use vs. never alcohol use), vigorous physical activity (yes vs. no), body mass index ([BMI] underweight, normal, overweight, or obesity), and self-reported health (excellent/very good, good, fair/poor). Finally, self-reported comorbidities were included as individual covariates (hypertension, diabetes, lung disease, heart disease, stroke, and arthritis) [1, 14, 32]. All covariates were collected at baseline as time-fixed variables.

### Statistical analysis

We examined changes in IADL independence (binary) over time in relation to cancer onset using a linear probability model with repeated measures (i.e., generalized estimating equations [GEE]) with age as the time scale. This approach utilized an identity link function and robust variance estimators to ensure validity of standard errors [33]. While this method can potentially yield predicted probabilities outside the range of 0 to 1, estimated risk differences are clearer to communicate than odds ratios derived from logistic regressions [32]. Our modeling strategy followed a previously published approach [34, 35], with the model specification:

$$\begin{aligned}{y}_{ti}={\beta }_{0}+{\beta }_{1}&{\left(interview \ age-75\right)}_{ti}+{\beta }_{2}{(interview \ age-75)}_{ti}^{2}+{\beta }_{3}{\left(ever \ cancer\right)}_{i}\\& \ \ \ \ \ \ +{\beta }_{4}{\left(age \ at \ cancer-75\right)}_{i}+{\beta }_{5}{\left(years \ before \ cancer \ diagnosis\right)}_{ti}+{\beta }_{6}{\left(cancer \ now\right)}_{ti}\\& \ \ \ \ \ \ +{\beta }_{7}{\left(years \ after \ cancer \ diagnosis\right)}_{ti}+\sum_{k}{\beta }_{8}{\left(covariates\right)}_{ki}\end{aligned}$$

The model included both individuals who were diagnosed with cancer and those who remained cancer-free. The outcome, ${y}_{ti}$, represented the probability that individual i was independent in IADL at time t. For cancer-free individuals, the “interview age” was centered at 75 years, with the linear (${\beta }_{1}$) and quadratic (${\beta }_{2}$) age terms capturing functional change with aging. The quadratic term accounted for curvilinear trajectories of functional status in older adults [36].

For individuals diagnosed with cancer, a different set of predictors was used to account for time relative to cancer diagnosis and the “interview age” was set to 0. The coefficient ${\beta }_{3}$ captured the overall difference in IADL independence between individuals ever diagnosed with cancer and those who were not. The coefficient ${\beta }_{4}$ reflected the effect of age at cancer diagnosis, centered at 75 years. “Years before cancer diagnosis” and “years after cancer diagnosis” were the difference between each participant’s age at each interview and their age at cancer diagnosis, with ${\beta }_{5}$ and ${\beta }_{7}$ representing the annual rate of change in independence before and after cancer diagnosis. Additionally, ${\beta }_{6}$ captured the immediate change in IADL independence around the time of cancer onset, with the predictor “cancer now” set to 0 before diagnosis and 1 after diagnosis.

The model was adjusted for relevant covariates including gender, race, educational attainment, marital status, total household wealth, tobacco use, alcohol use, vigorous physical activity, BMI, self-reported health, and self-reported comorbidities. We evaluated model fit by comparing models with and without the quadratic age term using the quasi-likelihood under the independence model criterion (QIC) and QIC_u_ based on GEE [37].

Our primary comparisons included differences in predicted probabilities of IADL independence between individuals with and without cancer diagnosis, and changes in the annual rate of IADL independence before and after cancer diagnosis among those with cancer. Predicted probabilities of IADL independence were computed under three scenarios: (1) never diagnosed with cancer; (2) before cancer onset; and (3) after cancer onset. These predictions were generated at various time points around age 75 (e.g., 5 years before and after diagnosis). In addition, we conducted stratified analyses by cancer site (i.e., prostate, lung, breast, and colorectal) to explore potential heterogeneity in the relationship between cancer and functional decline.

All models incorporated 1998 survey sampling weights to ensure representativeness of the 1998 US population born in 1949 or earlier [25]. Using the same methodological approach, we also assessed probabilities of independence in ADL, mobility, large muscle, gross motor, and fine motor skills. All analyses were performed using Stata/SE Version 18.0 (RRID:SCR_012763).

## Results

The study included a total of 15,972 participants with an average age of 66.1 years (SD = 9.9) at baseline (Table 1). The majority of the sample were female (57.3%), White (82.2%), non-Hispanic (92.0%), and married (68.4%). Throughout follow-up, 3,688 participants were diagnosed with cancer, and 12,284 participants remained cancer-free. The average follow-up was 14.0 years (SD = 7.02), and the mean age at cancer diagnosis was 74.7 years (SD = 8.67). Of the 15,972 participants, 9,386 reported deaths during the study period. Among those who died, 26.5% reported having been diagnosed with cancer before their death. A comparison of demographic characteristics between HRS participants with or without complete baseline data is presented in Supplementary Table S3.

**Table 1:** Baseline characteristics of study population by cancer status during follow-up

	Never cancer (*N* = 12,284)	Ever cancer (*N* = 3,688)	Total (*N* = 15,972)
	*N* (%)	*N* (%)	*N* (%)
**Demographic variables**			
Follow-up years, years^a^	13.9 (7.2)	14.5 (6.5)	14.0 (7.02)
Age at baseline, years^a^	66.39 (10.25)	65.13 (8.69)	66.10 (9.93)
Female	7,379 (60.1%)	1,774 (48.1%)	9,153 (57.3%)
Race			
White	10,038 (81.7%)	3,092 (83.8%)	13,130 (82.2%)
Black/African American	1,773 (14.4%)	507 (13.7%)	2,280 (14.3%)
Other	473 (3.9%)	89 (2.4%)	562 (3.5%)
Hispanic	1,067 (8.7%)	215 (5.8%)	1,282 (8.0%)
Educational attainment (college and above)	4,371 (35.6%)	1,437 (38.6%)	5,808 (36.3%)
Marital status at baseline			
Married	8,267 (67.3%)	2,656 (72.0%)	10,923 (68.4%)
Separated/divorced	1,214 (9.9%)	391 (10.6%)	1,605 (10.0%)
Widowed	2,441 (19.9%)	557 (15.1%)	2,998 (18.8%)
Never Married	362 (2.9%)	84 (2.3%)	446 (2.8%)
Total household wealth, per $1,000^a^	50.16 (111.04)	55.04 (73.07)	51.28 (103.53)
**Health-related variables**			
Ever smoking	6,870 (55.9%)	2,448 (66.4%)	9,318 (58.3%)
Ever alcohol use	7,419 (60.4%)	2,524 (68.4%)	9,943 (62.3%)
Vigorous physical activity	5,372 (43.7%)	1,743 (47.3%)	7,115 (44.5%)
BMI Category			
Underweight (< 18.5)	4,355 (35.5%)	1,209 (32.8%)	5,564 (34.8%)
Normal (18.5–24.9)	241 (2.0%)	47 (1.3%)	288 (1.8%)
Overweight/Obesity (> = 25.0)	7,688 (62.6%)	2,453 (66.0%)	10,141 (63.4%)
Self-reported health			
Excellent/very good	4,896 (39.9%)	1,612 (43.5%)	6,508 (40.7%)
Good	3,821 (31.1%)	1,152 (31.2%)	4,973 (31.1%)
Fair/poor	3,567 (29.0%)	942 (25.4%)	4,509 (36.7%)
**Health conditions**			
High blood pressure	5,226 (42.5%)	1,493 (40.5%)	6,719 (42.1%)
Diabetes	1,595 (13.0%)	394 (10.7%)	1,989 (12.5%)
Lung disease	700 (5.7%)	258 (7.0%)	958 (6.0%)
Heart disease	2,274 (18.5%)	631 (17.1%)	2,905 (18.2%)
Stroke	773 (6.3%)	197 (5.3%)	970 (6.1%)
Psychiatric problems	1,147 (9.3%)	294 (8.0%)	1,441 (9.0%)
Arthritis	5,822 (47.4%)	1,686 (45.7%)	7,508 (47.0%)

Abbreviation: *BMI* body mass index

^a^Mean (standard division) is reported for a continuous variable

**Fig. 2:**
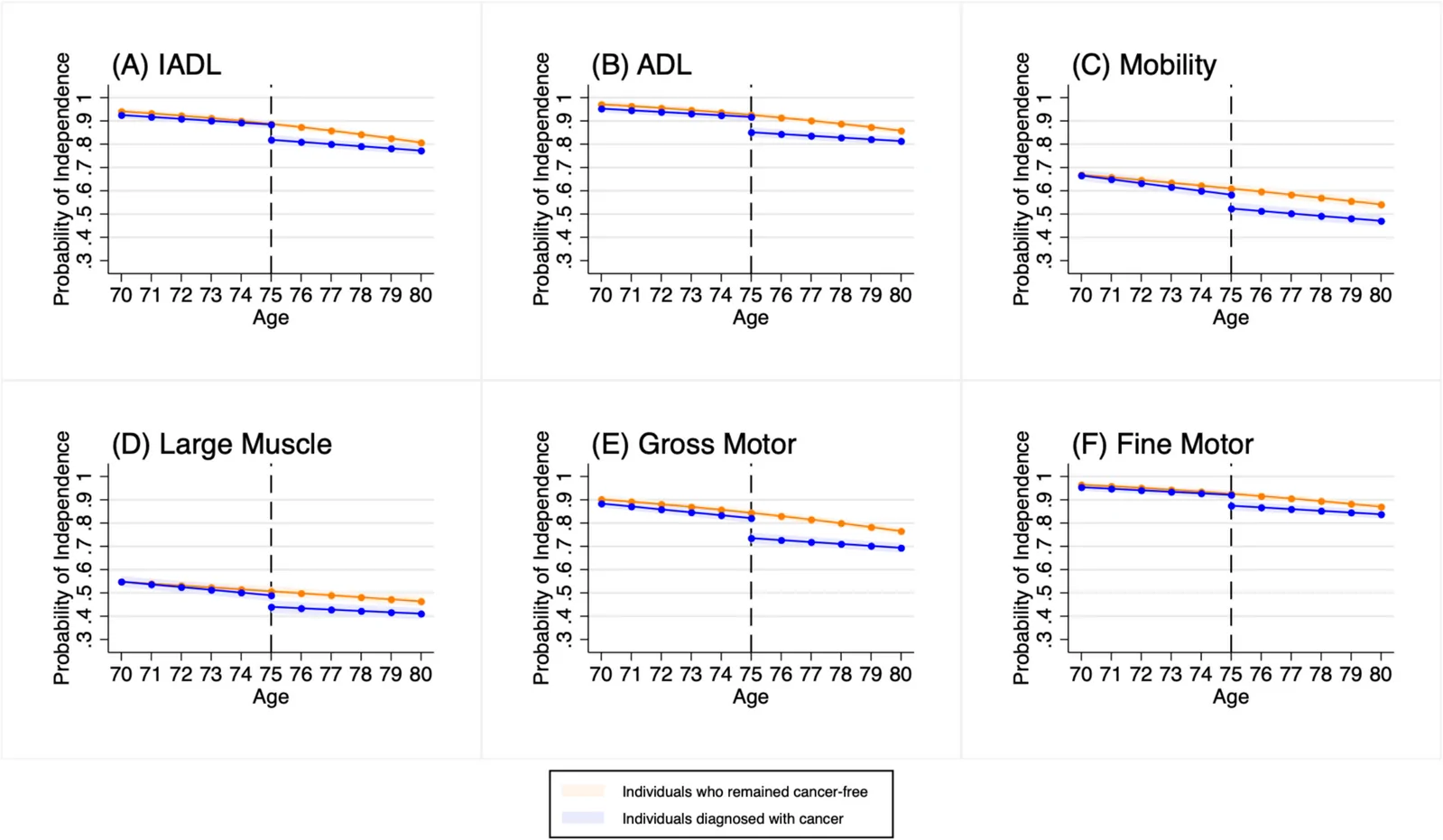
The predicted probability and 95% confidence intervals of independence by cancer status. Predicted probabilities of independence and 95% confidence intervals comparing individuals diagnosed with cancer at age 75 with those who remained cancer-free. (**A**) IADL; (**B**) ADL; (**C**) mobility; (**D**) large muscle; (**E**) gross motor; (**F**) fine motor. Predictions were generated for a 75-year-old married, non-Hispanic white woman who had not attained high school education, had no comorbidities, did not smoke, did not consume alcohol, had a normal body mass index, and reported excellent or very good self-reported health

Figure 2 illustrates predicted probabilities of functional independence (no difficulty performing the specified tasks) for individuals with and without cancer, assuming cancer onset at age 75 and adjusting for all covariates. Among individuals who remained cancer-free, 88.7% were estimated to have IADL independence at age 75 (Table 2). In this group, the initial rate of decline in IADL independence was 1.3% per year (95% CI =  − 1.4, − 1.3). However, this decline accelerated as individuals age, with an additional 0.1% reduction in IADL independence each year (95% CI =  − 0.1, − 0.1). Immediately before cancer diagnosis, the difference in probability of IADL independence between individuals with and without cancer at age 75 was close to null (− 0.3%, 95% CI =  − 0.4, 0.3). However, the probability of IADL independence dropped by 6.6% (95% CI =  − 8.1, − 5.0) immediately following cancer diagnosis at age 75.

**Table 2:** Estimates of probability of functional independence and differences in probability of functional independence by cancer status using generalized estimating equations (GEE)

	IADL		ADL		Mobility		Large muscle		Gross motor		Fine motor	
Characteristics	${\boldsymbol{\beta}}$(%)	95% CI	${\boldsymbol{\beta}}$(%)	95% CI	${\boldsymbol{\beta}}$(%)	95% CI	${\boldsymbol{\beta}}$(%)	95% CI	${\boldsymbol{\beta}}$(%)	95% CI	${\boldsymbol{\beta}}$(%)	95% CI
Functional independence at age 75 (intercept${\beta }_{0}$)	88.7	87.5, 90.0	92.6	91.3, 93.8	61.0	59.1, 62.9	50.7	48.8, 52.6	84.4	83.0, 85.8	92.6	91.3, 93.8
Participants with no cancer diagnosis during follow-up												
Linear change with age (${\beta }_{1}$)	− 1.3	− 1.4, − 1.3	− 1.1	− 1.2, − 1.1	− 1.3	− 1.3, − 1.2	− 0.8	− 0.9, − 0.8	− 1.4	− 1.4, − 1.3	−0.9	−1.0, −0.9
Non-linear change with age (${\beta }_{2}$)	− 0.1	− 0.1, − 0.1	0.0	0.0, 0.0	0.0	0.0, 0.0	0.0	0.0, 0.0	0.0	0.0, 0.0	0.0	0.0, 0.0
Participant with cancer diagnosis during follow-up												
Indicator for cancer diagnosis (${\beta }_{3}$)	− 0.3	− 1.5, 1.0	− 0.9	− 2.2, 0.5	− 2.7	− 4.4, − 1.0	− 1.7	− 3.4, 0.1	− 2.3	− 3.8, − 0.8	− 0.5	− 1.7, 0.8
Age at cancer diagnosis, centered at 75 (${\beta }_{4}$)	− 0.8	− 0.8, − 0.7	− 0.6	− 0.7, − 0.5	− 0.9	− 1.0, − 0.8	− 0.6	− 0.7, − 0.5	− 0.8	− 0.9, − 0.7	− 0.5	− 0.6, − 0.4
Rate of change before cancer diagnosis (${\beta }_{5}$)	− 0.8	− 0.9, − 0.7	− 0.7	− 0.8, − 0.6	− 1.7	− 1.8, − 1.5	− 1.2	− 1.3, − 1.0	− 1.3	− 1.4, − 1.1	− 0.7	− 0.8, − 0.5
Acute change at cancer diagnosis (${\beta }_{6}$)	− 6.6	− 8.1, − 5.0	− 6.6	− 8.3, − 4.9	− 5.9	− 7.8, − 4.0	− 5.0	− 6.8, − 3.1	− 8.6	− 10.4, − 6.8	− 4.6	− 6.2, − 3.1
Rate of change after cancer diagnosis (${\beta }_{7}$)	− 0.9	− 1.2, − 0.7	− 0.8	− 1.0, − 0.5	− 1.1	− 1.3, − 0.8	− 0.6	− 0.8, − 0.3	− 0.8	− 1.1, − 0.6	− 0.7	− 1.0, − 0.5

Abbreviation: *IADL* instrumental activities of daily living, *ADL* activities of daily living, *CI* confidence interval

Note: Values within ± 0.01% are shown as 0.0% to maintain one decimal place

Additionally, the prevalence of IADL independence was compared between individuals with and without cancer, using the coefficients from the fully adjusted model in Table 2. Before cancer diagnosis, individuals who developed cancer after age 75 had a lower probability of IADL independence in the 5 years leading up to diagnosis compared to those who remained cancer-free, with a small difference of 1.3% (95% CI =  − 1.9, − 0.7) between the groups. In contrast, there was a shift in the magnitude of the difference in probability of IADL independence following cancer diagnosis when compared to the cancer-free group. Individuals diagnosed with cancer experienced a 3.4% (95% CI =  − 4.6, − 2.2) lower probability of IADL independence over the subsequent 5 years shortly after diagnosis compared to their cancer-free counterparts.

The results for ADL independence exhibited similar patterns in terms of magnitude and direction of change. Compared to ADL and IADL independence, probabilities of independence at baseline for *mobility* and *large muscle* domains were lower (50.7% and 61.0%, respectively) (Table 2). The most substantial decrease in probability of independence immediately following cancer diagnosis was in the *gross motor* domain (walking one block, walking across the room, climbing one flight of stairs, and bathing), with an estimated reduction of 8.6% (95% CI =  − 10.4, − 6.8). The results regarding the probability of independence in individual tasks showed that activities such as walking several blocks, walking one block, climbing several stairs, and climbing one stair were most affected immediately following cancer diagnosis, with reductions ranging from 6.3% to 9.6% (Supplementary Tables S4-S9).

To further examine the relationship between cancer and functional decline, we stratified cancer into subgroups of the most common cancer sites in our study sample: prostate, lung, breast, and colorectal, which accounted for 20.0%, 17.2%, 13.3%, and 11.0% of the reported cancer sites (*N* = 3,308), respectively (Supplementary Table S10). In stratified analyses, similar patterns were observed across sites, with varying degrees of probability of independence. For instance, loss of independence immediately following diagnosis was greatest for individuals diagnosed with lung cancer (−11.5% for IADL independence (95% CI =  − 17.1, − 5.9) and − 12.8% for ADL independence (95% CI =  − 18.9, − 6.8)) (Supplementary Tables S11-S12). These findings underscore the severe prognosis associated with lung cancer.

## Discussion

In this study, we examined the long-term trajectories of functional limitation in individuals with cancer compared to those who remained cancer-free over an average follow-up period of 14 years. Our findings indicate a sharp initial drop in the probability of functional independence immediately following cancer diagnosis. However, individuals with cancer experience a similar or slower rate of functional decline compared to those without cancer after the post-diagnosis period.

Our results of the short-term functional decline immediately following cancer diagnosis align with earlier studies conducted among older adults around the time of cancer diagnosis or during active treatment [1]. This initial reduction in functional status appears to be attributed to the effects of cancer treatment. However, detailed information on the first course of treatment modality would be required to fully understand the impact of cancer treatment. A few other studies have demonstrated that certain cancer treatments, such as chemotherapy-related toxicity and hormone therapy, may have a pronounced impact on early functional decline during treatment for older patients [21, 22, 31], associated with severe side effects including nausea, vomiting, weakness, and fatigue [38]. Older cancer patients who undergo surgery experience fewer permanent functional limitations and are more likely to recover to baseline functional status within 3 months of treatment completion, although recovery periods vary [18, 20]. However, previous research on functional decline among cancer patients has primarily captured specific time points following cancer diagnosis, comparing cancer patients’ functional status at one or two follow-up assessments to their baseline level, rather than looking at trajectories over a longer period. Additionally, this research is often limited to the first-year post-diagnosis or treatment initiation [16, 17, 22, 30–32, 39]. Thus, evidence from our longitudinal study on functional decline combined with previous research provides a more comprehensive understanding of how functional status changes over time for adults aged 50 years and older with cancer compared to their cancer-free counterparts. As suggested by the accelerated aging model [1, 12], we observed that cancer incidence and treatment appear to cause a short-term deviation from the expected trajectory of normal aging around the time of diagnosis. However, the functional limitation among older adult cancer survivors does not continue at an accelerated rate over the long term.

Despite the recommendations for integrating functional assessments into routine oncology care [40], their implementation may have been hindered by resource constraints and a perceived lack of utility among oncology care providers [41]. To alleviate these barriers, understanding trajectories of functional limitation prior to and following cancer diagnosis becomes crucial for effectively allocating and prioritizing resources for older adult cancer survivors. Our findings corroborate evidence from a longitudinal study in the Chinese population, indicating a minimal difference in the overall trajectory of functional limitation between individuals with and without cancer, despite the use of different functional assessment measures than ADL and IADL [15]. Building upon these insights, we extended our study by not only examining functional decline before and after cancer diagnosis among individuals with cancer [15], but also comparing these trajectories to those of individuals who remained cancer-free. This approach allowed us to evaluate the impact of cancer at different points in time. Contrary to the pre-diagnosis findings, individuals with cancer experienced a slower functional decline after a sharp drop in functional status immediately following diagnosis compared to their cancer-free counterparts. This observation could reflect the consistent care provided to older adult cancer survivors [42, 43], irrespective of whether care plans specifically address functional limitations. In contrast, cancer-free individuals may not have regular clinical interactions to detect early indications of functional limitations, potentially leading to a steeper decline later in life [44]. The slower functional decline in individuals with cancer following diagnosis in the current study is a positive development. Yet, the integration of routine functional assessments into oncology care is important for directly managing functional limitations, which can help mitigate further decline and the numerous consequences of functional loss in older adults. The integration can be enhanced by strategically allocating assessments at different time points. Our findings indicate that functional assessments remain crucial predictors of susceptibility to treatment-related toxicity before treatment initiation [6, 9, 11]. Moreover, even infrequent assessments after treatment completion are necessary to detect subtle changes in function and allow for early intervention to prevent further deterioration. Future studies should examine whether the slower rate of functional decline observed during the post-diagnosis period is associated with the frequency of healthcare utilization, as its exact role in alleviating functional decline remains unclear.

Given that the number of cancer survivors reporting functional limitations has more than doubled in the last two decades [4], the current results highlight the importance of integrating functional assessments within the broader context of geriatric care to improve quality of life in older adult cancer survivors. Addressing functional limitations alone is insufficient for improving quality of life, as functional decline is strongly associated with cognitive deficits, another key component of geriatric assessment, and dementia in older adults [45]. Research examining cognitive memory decline in cancer patients indicates that individuals with cancer may retain better memory function and experience a slower memory decline than cancer-free individuals over time [35]. Moreover, a joint analysis of ADL and memory loss using group-based trajectory modeling reveals that individuals with higher levels of memory retention experience fewer ADL limitations and vice versa among cancer survivors [46]. These findings, alongside our observation of functional decline in cancer patients compared to their cancer-free counterparts, can be utilized to create treatment plans tailored to the specific needs of older adult cancer survivors. Furthermore, they can inform the development of more comprehensive interventions aimed at maintaining quality of life and addressing frailty for individuals in the cancer care continuum.

While our study provides valuable insights, it is not without limitations. First, all measures were based on self-reports from HRS participants, which may introduce recall bias for certain measures and an issue of incomplete responses. However, self-reported cancer diagnoses have been shown to be reasonably accurate when validated against Medicare records, with 73.2% sensitivity and 96.2% specificity for the first cancer incident reported between 2000 and 2016 [47]. Moreover, as a population-based study, the HRS provides a better generalizability to the overall population of US older adults compared to other study designs. Given the limited and potentially inaccurate data on cancer type and treatment modalities [47], it remains unclear how various cancer-related factors impact functional decline. We also focused our analysis on HRS participants who were alive and well enough to participate in study interviews at the time of data collection, a limitation shared by other research in this area. This may have led to an underestimation of the total effect of cancer on functional decline due to the exclusion of those who died soon after being diagnosed. However, the number of deaths was comparable between those with and without cancer, suggesting that the bias introduced by excluding deceased participants is likely minimal. Additionally, we examined functional status as a binary variable for each functional domain assessed. This approach was chosen for its interpretability and due to a small number of participants reporting difficulties in performing two or more tasks per functional domain. The low number of participants reporting difficulties in multiple tasks may reflect attrition of participants who were severely ill to respond. Future studies could address this limitation by considering the use of proxy respondents to report on the functional status of participants who are unable to respond due to severe illness. Finally, the variability in functional measurements introduces a challenge in comparing results across different studies. This variability includes differences in item selection, scoring methods, and the type of respondent. While we included other measurements (i.e., mobility, large muscle, gross motor, and fine motor skills) with ADL and IADL to minimize this gap, standardizing the functional measurements would significantly enhance the comparability and interpretation of the findings.

In conclusion, in this analysis, cancer patients did not experience a decline that substantially diverged from the normal aging trajectory prior to the onset of cancer diagnosis. Additionally, the slower rate of functional decline during the post-diagnosis period indicates a potential for recovery or stabilization among cancer patients following the diagnosis. However, the sharp decline in function observed immediately after cancer diagnosis represents a critical window for clinical and caregiver preparation, underscoring the need for timely interventions to support cancer patients during this vulnerable period. Our study contributes to a growing body of literature highlighting the crucial role of functional assessment for older adults diagnosed with cancer, particularly emphasizing the critical timing around the cancer diagnosis. While our focus was solely on functional limitations, research exploring the complex interplay between cancer and various domains of geriatric assessment, including functional and cognitive domains, merits further clinical attention and action. By addressing these gaps, healthcare providers can better allocate resources and conduct geriatric assessments to effectively manage the overall well-being of older adults affected by cancer.

## Supplementary Information

Below is the link to the electronic supplementary material.

ESM 1 (DOCX 52.9 KB)

## Data Availability

The HRS (RRID:SCR_008930) is sponsored by the National Institute on Aging (grant number NIA U01AG009740) and is conducted by the University of Michigan (RRID:SCR_011668). The data product, RAND HRS Longitudinal File 2020 (V1), is a publicly available dataset. Available from: https://hrs.isr.umich.edu/data-products. In addition, the data on cancer sites from the HRS are available upon reasonable request from the corresponding author.
