# Correction: The impact of cancer diagnosis on functional decline in adults aged 50 and older: the US Health and Retirement Study

**DOI:** 10.1007/s11764-025-01884-4

**Published:** 2025-08-28

**Authors:** Gina E. Nam, Elizabeth Rose Mayeda, Yancen Pan, Eleanor Hayes-Larson, L. Paloma Rojas-Saunero, Hua Zhou, Jian Yu Rao, Zuo-Feng Zhang

**Affiliations:** 1 Department of Epidemiology, UCLA Fielding School of Public Health, 71-225 CHS, 650 Charles E. Young Drive South, Box 951772, Los Angeles, CA 90095-1772 USA; 2 Leonard Davis School of Gerontology, University of Southern California, Los Angeles, CA USA; 3 Department of Biostatistics, UCLA Fielding School of Public Health, Los Angeles, CA USA; 4 Department of Computational Medicine, UCLA David Geffen School of Medicine, Los Angeles, CA USA; 5 Department of Pathology and Laboratory Medicine, UCLA David Geffen School of Medicine, Los Angeles, CA USA; 6 UCLA Jonsson Comprehensive Cancer Center, Los Angeles, CA USA; 7 Department of Medicine, Center for Human Nutrition, UCLA David Geffen School of Medicine, Los Angeles, CA USA

**Correction: Journal of Cancer Survivorship**

10.1007/s11764-025-01867-5

In this article, the author’s name Jian Yu Rao was incorrectly written as JianJian Yu Rao.

The original article has been corrected.

