# Supplementary material for: The impact of cancer diagnosis on functional decline in adults aged 50 and older: the US Health and Retirement Study
Source: J Cancer Surviv. 2025 Jul 29;20(Suppl 1):881–91. doi: 10.1007/s11764-025-01867-5 (PMC13131248; doi:10.1007/s11764-025-01867-5)
Supplement: Supplementary file 1 [file 11764_2025_1867_MOESM1_ESM.docx]

**Supplementary Information**

**Title:**

The impact of cancer diagnosis on functional decline in older cancer survivors: the U.S. Health and Retirement Study

**Authors and affiliations:**

Gina E. Nam^1^; Elizabeth Rose Mayeda^1^; Yancen Pan^1^; Eleanor Hayes-Larson,^2^; L. Paloma Rojas-Saunero^1^; Hua Zhou^3,4^; Jian Yu Rao^1,5^; Zuo-Feng Zhang^1,6,7*^

^1^Department of Epidemiology, UCLA Fielding School of Public Health, Los Angeles, California, USA

^2^USC Davis School of Gerontology, Los Angeles, CA

^3^Department of Biostatistics, University of California, Los Angeles, California, USA

^4^Department of Computational Medicine, David Geffen School of Medicine, University of California, Los Angeles, California, USA

^5^Department of Pathology and Laboratory Medicine, UCLA David Geffen School of Medicine, Los Angeles, California, USA

^6^Jonsson Comprehensive Cancer Center, UCLA, Los Angeles, California, USA

^7^Department of Medicine, Center for Human Nutrition, UCLA David Geffen School of Medicine, Los Angeles, California, USA

**Corresponding Author:**

Zuo-Feng Zhang

Mailing address: Department of Epidemiology, UCLA Fielding School of Public Health, 71-225 CHS, Box 951772, 650 Charles E. Young Drive South, Los Angeles, California 90095-1772, USA

E-mail address: [zfzhang@ucla.edu](mailto:zfzhang@ucla.edu)

**Table of contents:**

**Supplementary Table S1**. Incident cancer ascertainment (N = 3,688)

**Supplementary Table S2**. Description of each index

**Supplementary Table S3**. Comparison of demographic characteristics between study participants with complete baseline data and those with missing information

**Supplementary Table S4**. Estimates of Probability of IADL Independence and Differences in Probability of IADL Independence by Cancer Status

**Supplementary Table S5**. Estimates of Probability of ADL Independence and Differences in Probability of ADL Independence by Cancer Status

**Supplementary Table S6**. Estimates of Probability of Mobility Independence and Differences in Probability of Mobility Independence by Cancer Status

**Supplementary Table S7**. Estimates of Probability of Large Muscle Independence and Differences in Probability of Large Muscle Independence by Cancer Status

**Supplementary Table S8.** Estimates of Probability of Gross Motor Independence and Differences in Probability of Gross Motor Independence by Cancer Status

**Supplementary Table S9**. Estimates of Probability of Fine Motor Independence and Differences in Probability of Fine Motor Independence by Cancer Status

**Supplementary Table S10**. Distribution of Cancer Sites (Excluding Non-Melanoma Skin Cancer)

**Supplementary Table S11.** Estimates of Probability of IADL Independence and Differences in Probability of IADL Independence by Cancer Sites

**Supplementary Table S12**. Estimates of Probability of ADL Independence and Differences in Probability of ADL Independence by Cancer Sites

**Supplementary Table S1.** Incident cancer ascertainment (N = 3,688)

| Category | N (%) |
| --- | --- |
| Self-reported cancer diagnosis during follow-up interviews | 2,578 (69.9) |
| Proxy-reported cancer diagnosis from after-death interviews | 897 (24.3) |
| Cancer diagnosis date reported, but no report on having cancer diagnosis | 157 (4.3) |
| Cancer diagnosis reported, but no report on cancer diagnosis date | 56 (1.5) |

**Supplementary Table S2.** Description of each index

| **Index** | **Tasks** |
| --- | --- |
| ADL | bathing, eating, dressing, walking across a room, and getting in or out of bed |
| IADL | using a telephone, taking medication, handling money, shopping, preparing meals |
| Mobility | walking several blocks, walking one block, walking across the room, climbing several flights of stairs and climbing one flight of stairs. |
| Large muscle | sitting for two hours, getting up from a chair, stooping or kneeling or crouching, and pushing or pulling a large object |
| Gross motor | walking one block, walking across the room, climbing one flight of stairs, and bathing |
| Fine motor | picking up a dime, eating, and dressing |

**Supplementary Table S3.** Comparison of demographic characteristics between study participants with complete baseline data and those with missing information

|  | **Complete** | **Missing** |
| --- | --- | --- |
| **Characteristics** | **N (%)** | **N (%)** |
| Total | 15,972 | 560 |
| Age at baseline, years^a^ | 66.10 (9.93) | 60.3 (11.3) |
| Missing |  | 183 |
| Female | 9,153 (57.3%) | 425 (75.9%) |
| Race |  |  |
| White | 13,130 (82.2%) | 434 (78.1%) |
| Black | 2,280 (14.3%) | 87 (15.7%) |
| Other | 562 (3.5%) | 35 (6.3%) |
| Missing |  | 4 |
| Hispanic | 1,282 (8. ) | 81 (14.5%) |
| Missing |  | 2 |
| Educational attainment (College and above) | 5,808 (36.3%) | 195 (34.8%) |
| Missing |  | 1 |
| Marital status at baseline |  |  |
| Married | 10,923 (68.4%) | 434 (80.5%) |
| Separated/divorced | 1,605 (10. ) | 33 (6.1%) |
| Widowed | 2,998 (18.8%) | 57 (10.6%) |
| Never Married | 446 (2.8%) | 15 (2.8%) |
| Missing |  | 21 |
| Total household wealth, per $1,000^a^ | 51.28 (103.53) | 63.7 (160.6) |

^a^Mean (standard deviation) is reported for a continuous variable.

**Supplementary Table S4.** Estimates of Probability of IADL Independence and Differences in Probability of IADL Independence by Cancer Status

|  | **Bathing** | | **Eating** | | **Dressing** | | **Walking across a room** | | **Getting in and out of bed** | |
| --- | --- | --- | --- | --- | --- | --- | --- | --- | --- | --- |
| **Characteristics** | $\boldsymbol{\beta}$ **(%)** | **95% CI** | $\boldsymbol{\beta}$ **(%)** | **95% CI** | $\boldsymbol{\beta}$ **(%)** | **95% CI** | $\boldsymbol{\beta}$ **(%)** | **95% CI** | $\boldsymbol{\beta}$ **(%)** | **95% CI** |
| **Intercept** | 95.7 | 94.8, 96.6 | 97.2 | 96.5, 97.9 | 96.4 | 95.4, 97.4 | 96.6 | 95.7, 97.5 | 97.5 | 96.6, 98.3 |
| **Never cancer: age, centered at 75** | -0.9 | -1.0, -0.9 | -0.5 | -0.5, -0.5 | -0.8 | -0.8, -0.7 | -0.8 | -0.8, -0.7 | -0.5 | -0.5, -0.4 |
| **Never cancer: age, centered at 75 (quadratic)** | 0.0 | 0.0, 0.0 | 0.0 | 0.0, 0.0 | 0.0 | 0.0, 0.0 | 0.0 | 0.0, 0.0 | 0.0 | 0.0, 0.0 |
| **Cancer: Indicator** | -0.1 | -1.0, 0.8 | 0.3 | -0.3, 0.9 | -0.3 | -1.4, 0.8 | -0.4 | -1.3, 0.5 | 0.1 | -0.7, 0.9 |
| **Cancer: age at cancer, centered at 75** | -0.5 | -0.5, -0.4 | -0.2 | -0.3, -0.2 | -0.4 | -0.5, -0.3 | -0.4 | -0.5, -0.3 | -0.1 | -0.2, -0.1 |
| **Cancer: years before cancer** | -0.4 | -0.5, -0.3 | -0.2 | -0.2, -0.1 | -0.4 | -0.5, -0.3 | -0.4 | -0.5, -0.3 | -0.1 | -0.2, 0 |
| **Cancer: cancer now** | -4.2 | -5.3, -3 | -2.6 | -3.4, -1.8 | -3.9 | -5.3, -2.5 | -4.3 | -5.5, -3.1 | -3.2 | -4.3, -2.1 |
| **Cancer: years after cancer** | -0.7 | -0.9, -0.5 | -0.3 | -0.4, -0.2 | -0.6 | -0.8, -0.4 | -0.6 | -0.8, -0.4 | -0.3 | -0.5, -0.1 |

**Note**: Values within ±0.01% are shown as 0.0% to maintain one decimal place.

**Supplementary Table S5.** Estimates of Probability of ADL Independence and Differences in Probability of ADL Independence by Cancer Status

|  | **Using a telephone** | | **Taking medication** | | **Handling money** | | **Shopping** | | **Preparing meals** | |
| --- | --- | --- | --- | --- | --- | --- | --- | --- | --- | --- |
| **Characteristics** | $\boldsymbol{\beta}$ **(%)** | **95% CI** | $\boldsymbol{\beta}$ **(%)** | **95% CI** | $\boldsymbol{\beta}$ **(%)** | **95% CI** | $\boldsymbol{\beta}$ **(%)** | **95% CI** | $\boldsymbol{\beta}$ **(%)** | **95% CI** |
| **Intercept** | 95.1 | 94.3, 95.9 | 96.1 | 95.4, 96.8 | 93.7 | 92.8, 94.5 | 92.3 | 91.2, 93.3 | 92.9 | 92, 93.9 |
| **Never cancer: age, centered at 75** | -0.8 | -0.9, -0.8 | -0.5 | -0.6, -0.5 | -1.1 | -1.1, -1 | -1.2 | -1.2, -1.1 | -1.1 | -1.1, -1.1 |
| **Never cancer: age, centered at 75 (quadratic)** | 0.0 | 0.0, 0.0 | 0.0 | 0.0, 0.0 | 0.0 | 0.0, 0.0 | -0.1 | -0.1, 0 | 0.0 | 0.0, 0.0 |
| **Cancer: Indicator** | 0.2 | -0.6, 0.9 | 0.3 | -0.3, 0.9 | 0.0 | -0.9, 0.9 | -0.5 | -1.6, 0.6 | 0.1 | -0.8, 1 |
| **Cancer: age at cancer, centered at 75** | -0.4 | -0.5, -0.4 | -0.2 | -0.3, -0.2 | -0.5 | -0.6, -0.5 | -0.6 | -0.6, -0.5 | -0.5 | -0.6, -0.5 |
| **Cancer: years before cancer** | -0.3 | -0.4, -0.3 | -0.2 | -0.3, -0.1 | -0.4 | -0.5, -0.4 | -0.6 | -0.7, -0.5 | -0.5 | -0.6, -0.4 |
| **Cancer: cancer now** | -2.7 | -3.6, -1.7 | -2.6 | -3.6, -1.7 | -4.3 | -5.5, -3.2 | -5.3 | -6.7, -3.9 | -4.5 | -5.8, -3.3 |
| **Cancer: years after cancer** | -0.5 | -0.7, -0.4 | -0.3 | -0.5, -0.2 | -0.6 | -0.8, -0.5 | -0.8 | -1, -0.6 | -0.8 | -1, -0.6 |

**Note**: Values within ±0.01% are shown as 0.0% to maintain one decimal place.

**Supplementary Table S6.** Estimates of Probability of Mobility Independence and Differences in Probability of Mobility Independence by Cancer Status

|  | **Walking several blocks** | | **Walking one block** | | **Walking across the room** | | **Climbing several stairs** | | **Climbing one stair** | |
| --- | --- | --- | --- | --- | --- | --- | --- | --- | --- | --- |
| **Characteristics** | $\boldsymbol{\beta}$ **(%)** | **95% CI** | $\boldsymbol{\beta}$ **(%)** | **95% CI** | $\boldsymbol{\beta}$ **(%)** | **95% CI** | $\boldsymbol{\beta}$ **(%)** | **95% CI** | $\boldsymbol{\beta}$ **(%)** | **95% CI** |
| **Intercept** | 81.5 | 79.9, 83.1 | 92.0 | 90.8, 93.2 | 96.6 | 95.7, 97.5 | 60.5 | 58.6, 62.5 | 87.5 | 86.2, 88.8 |
| **Never cancer: age, centered at 75** | -1.5 | -1.5, -1.4 | -1.1 | -1.2, -1.1 | -0.8 | -0.8, -0.7 | -1.3 | -1.3, -1.2 | -1.1 | -1.1, -1 |
| **Never cancer: age, centered at 75 (quadratic)** | 0.0 | 0.0, 0.0 | 0.0 | 0.0, 0.0 | 0.0 | 0.0, 0.0 | 0.0 | 0.0, 0.0 | 0.0 | 0.0, 0.0 |
| **Cancer: Indicator** | -2.0 | -3.7, -0.4 | -1.3 | -2.6, 0 | -0.4 | -1.3, 0.5 | -2.1 | -3.9, -0.3 | -2.1 | -3.5, -0.8 |
| **Cancer: age at cancer, centered at 75** | -0.9 | -1, -0.8 | -0.7 | -0.8, -0.6 | -0.4 | -0.5, -0.3 | -0.8 | -0.9, -0.7 | -0.6 | -0.7, -0.5 |
| **Cancer: years before cancer** | -1.5 | -1.6, -1.3 | -1.0 | -1.1, -0.9 | -0.4 | -0.5, -0.3 | -1.5 | -1.7, -1.3 | -1.0 | -1.1, -0.8 |
| **Cancer: cancer now** | -9.6 | -11.4, -7.7 | -7.5 | -9.1, -5.9 | -4.3 | -5.5, -3.1 | -6.3 | -8.3, -4.3 | -7.0 | -8.6, -5.3 |
| **Cancer: years after cancer** | -0.8 | -1.1, -0.6 | -0.8 | -1, -0.5 | -0.6 | -0.8, -0.4 | -0.9 | -1.2, -0.7 | -0.7 | -0.9, -0.4 |

**Note**: Values within ±0.01% are shown as 0.0% to maintain one decimal place.

**Supplementary Table S7.** Estimates of Probability of Large Muscle Independence and Differences in Probability of Large Muscle Independence by Cancer Status

|  | **Sitting for 2 hours** | | **Getting up from a chair** | | **Stooping** | | **Pushing/pulling** | |
| --- | --- | --- | --- | --- | --- | --- | --- | --- |
| **Characteristics** | $\boldsymbol{\beta}$ **(%)** | **95% CI** | $\boldsymbol{\beta}$ **(%)** | **95% CI** | $\boldsymbol{\beta}$ **(%)** | **95% CI** | $\boldsymbol{\beta}$ **(%)** | **95% CI** |
| **Intercept** | 88.3 | 86.8, 89.7 | 74.9 | 73.1, 76.7 | 65.3 | 63.3, 67.2 | 74.3 | 72.6, 76 |
| **Never cancer: age, centered at 75** | 0.1 | 0.0, 0.1 | -0.7 | -0.7, -0.6 | -0.9 | -0.9, -0.8 | -1.1 | -1.1, -1 |
| **Never cancer: age, centered at 75 (quadratic)** | 0.0 | 0.0, 0.0 | 0.0 | 0.0, 0.0 | 0.0 | 0.0, 0.0 | 0.0 | 0.0, 0.0 |
| **Cancer: Indicator** | -0.3 | -1.7, 1.1 | -2.9 | -4.6, -1.2 | -1.2 | -2.9, 0.6 | -1.5 | -3.1, 0 |
| **Cancer: age at cancer, centered at 75** | 0.2 | 0.1, 0.3 | -0.4 | -0.6, -0.3 | -0.6 | -0.7, -0.5 | -0.5 | -0.6, -0.3 |
| **Cancer: years before cancer** | -0.1 | -0.2, 0.1 | -0.8 | -1, -0.6 | -1.2 | -1.3, -1 | -1.0 | -1.2, -0.9 |
| **Cancer: cancer now** | -2.1 | -3.7, -0.5 | -4.0 | -5.9, -2.1 | -3.6 | -5.6, -1.7 | -8.6 | -10.5, -6.6 |
| **Cancer: years after cancer** | 0.1 | -0.1, 0.3 | -0.3 | -0.5, -0.1 | -0.9 | -1.1, -0.6 | -0.4 | -0.6, -0.1 |

**Note**: Values within ±0.01% are shown as 0.0% to maintain one decimal place.

**Supplementary Table S8.** Estimates of Probability of Gross Motor Independence and Differences in Probability of Gross Motor Independence by Cancer Status

|  | **Walking one block** | | **Walking across the room** | | **Climbing one stair** | | **Bathing** | |
| --- | --- | --- | --- | --- | --- | --- | --- | --- |
| **Characteristics** | $\boldsymbol{\beta}$ **(%)** | **95% CI** | $\boldsymbol{\beta}$ **(%)** | **95% CI** | $\boldsymbol{\beta}$ **(%)** | **95% CI** | $\boldsymbol{\beta}$ **(%)** | **95% CI** |
| **Intercept** | 92.0 | 90.8, 93.2 | 96.6 | 95.7, 97.5 | 87.5 | 86.2, 88.8 | 95.7 | 94.8, 96.6 |
| **Never cancer: age, centered at 75** | -1.1 | -1.2, -1.1 | -0.8 | -0.8, -0.7 | -1.1 | -1.1, -1 | -0.9 | -1, -0.9 |
| **Never cancer: age, centered at 75 (quadratic)** | 0.0 | 0.0, 0.0 | 0.0 | 0.0, 0.0 | 0.0 | 0.0, 0.0 | 0.0 | 0.0, 0.0 |
| **Cancer: Indicator** | -1.3 | -2.6, 0 | -0.4 | -1.3, 0.5 | -2.1 | -3.5, -0.8 | -0.1 | -1, 0.8 |
| **Cancer: age at cancer, centered at 75** | -0.7 | -0.8, -0.6 | -0.4 | -0.5, -0.3 | -0.6 | -0.7, -0.5 | -0.5 | -0.5, -0.4 |
| **Cancer: years before cancer** | -1.0 | -1.1, -0.9 | -0.4 | -0.5, -0.3 | -1.0 | -1.1, -0.8 | -0.4 | -0.5, -0.3 |
| **Cancer: cancer now** | -7.5 | -9.1, -5.9 | -4.3 | -5.5, -3.1 | -7.0 | -8.6, -5.3 | -4.2 | -5.3, -3 |
| **Cancer: years after cancer** | -0.8 | -1, -0.5 | -0.6 | -0.8, -0.4 | -0.7 | -0.9, -0.4 | -0.7 | -0.9, -0.5 |

**Note**: Values within ±0.01% are shown as 0.0% to maintain one decimal place.

**Supplementary Table S9.** Estimates of Probability of Fine Motor Independence and Differences in Probability of Fine Motor Independence by Cancer Status

|  | **Picking up a dime** | | **Eating** | | **Dressing** | |
| --- | --- | --- | --- | --- | --- | --- |
| **Characteristics** | $\boldsymbol{\beta}$ **(%)** | **95% CI** | $\boldsymbol{\beta}$ **(%)** | **95% CI** | $\boldsymbol{\beta}$ **(%)** | **95% CI** |
| **Intercept** | 95.7 | 94.7, 96.6 | 97.2 | 96.5, 97.9 | 96.4 | 95.4, 97.4 |
| **Never cancer: age, centered at 75** | -0.4 | -0.5, -0.4 | -0.5 | -0.5, -0.5 | -0.8 | -0.8, -0.7 |
| **Never cancer: age, centered at 75 (quadratic)** | 0.0 | 0.0, 0.0 | 0.0 | 0.0, 0.0 | 0.0 | 0.0, 0.0 |
| **Cancer: Indicator** | 0.1 | -0.8, 1 | 0.3 | -0.3, 0.9 | -0.3 | -1.4, 0.8 |
| **Cancer: age at cancer, centered at 75** | -0.3 | -0.3, -0.2 | -0.2 | -0.3, -0.2 | -0.4 | -0.5, -0.3 |
| **Cancer: years before cancer** | -0.3 | -0.4, -0.2 | -0.2 | -0.2, -0.1 | -0.4 | -0.5, -0.3 |
| **Cancer: cancer now** | -2.2 | -3.4, -1.1 | -2.6 | -3.4, -1.8 | -3.9 | -5.3, -2.5 |
| **Cancer: years after cancer** | -0.4 | -0.6, -0.3 | -0.3 | -0.4, -0.2 | -0.6 | -0.8, -0.4 |

**Note**: Values within ±0.01% are shown as 0.0% to maintain one decimal place.

**Supplementary Table S10.** Distribution of Cancer Sites (Excluding Non-Melanoma Skin Cancer)^a^

| **Cancer Sites** | **N (%)** |
| --- | --- |
| Prostate | 661 (20.0) |
| Lung | 569 (17.2) |
| Breast | 441 (13.3) |
| Colorectal | 364 (11.0) |
| Other^b^ | 1,273 (38.5) |

^a^The total number of cancer cases in this table differs from the overall number of cancer diagnoses, as not all individuals reported specific cancer sites.

^b^Include 45 cancer sites, excluding prostate, lung, breast, and colorectal cancer.

**Supplementary Table S11.** Estimates of Probability of IADL Independence and Differences in Probability of IADL Independence by Cancer Sites

|  | **Prostate** |  | **Lung** |  | **Breast** |  | **Colorectal** |  |
| --- | --- | --- | --- | --- | --- | --- | --- | --- |
| **Characteristics** | $\boldsymbol{\beta}$ **(%)** | **95% CI** | $\boldsymbol{\beta}$ **(%)** | **95% CI** | $\boldsymbol{\beta}$ **(%)** | **95% CI** | $\boldsymbol{\beta}$ **(%)** | **95% CI** |
| Intercept | 88.5 | 87.2, 89.9 | 88.8 | 87.5, 90.2 | 88.6 | 87.3, 90.0 | 88.8 | 87.4, 90.2 |
| Never cancer: age, centered at 75 | -1.3 | -1.4, -1.3 | -1.4 | -1.4, -1.3 | -1.4 | -1.4, -1.3 | -1.3 | -1.4, -1.3 |
| Never cancer: age, centered at 75 (quadratic) | -0.1 | -0.1, -0.1 | -0.1 | -0.1, -0.1 | -0.1 | -0.1, -0.1 | -0.1 | -0.1, -0.1 |
| Cancer: indicator | 2.9 | 0.6, 5.3 | -0.7 | -4.2, 0.3 | -0.6 | -3.6, 2.5 | -2.4 | -6.2, 1.3 |
| Cancer: age at cancer, centered at 75 | -0.6 | -0.8, -0.4 | -0.4 | -0.7, -0.1 | -1.0 | -1.2, -0.8 | -0.8 | -1.2, -0.4 |
| Cancer: years before cancer | -0.4 | -0.6, -0.2 | -0.8 | -1.2, -0.3 | -0.8 | -1.1, -0.5 | -0.9 | -1.3, -0.5 |
| Cancer: cancer now | -2.1 | -4.9, 0.7 | -11.5 | -17.1, -5.9 | -2.9 | -6.1, 0.3 | -6.5 | -11.1, -1.8 |
| Cancer: years after cancer | -1.1 | -1.5, -0.8 | -0.7 | -1.7, 0.2 | -1.6 | -2.1, -1.1 | -1.2 | -1.9, -0.5 |

**Note**: Values within ±0.01% are shown as 0.0% to maintain one decimal place.

**Supplementary Table S12.** Estimates of Probability of ADL Independence and Differences in Probability of ADL Independence by Cancer Sites

|  | **Prostate** |  | **Lung** |  | **Breast** |  | **Colorectal** |  |
| --- | --- | --- | --- | --- | --- | --- | --- | --- |
| **Characteristics** | $\boldsymbol{\beta}$ **(%)** | **95% CI** | $\boldsymbol{\beta}$ **(%)** | **95% CI** | $\boldsymbol{\beta}$ **(%)** | **95% CI** | $\boldsymbol{\beta}$ **(%)** | **95% CI** |
| Intercept | 92.4 | 91.0, 93.7 | 92.6 | 91.2, 93.9 | 92.5 | 91.1, 93.8 | 92.6 | 91.3, 94.0 |
| Never cancer: age, centered at 75 | -1.1 | -1.2, -1.1 | -1.1 | -1.2, -1.1 | -1.1 | -1.2, -1.1 | -1.1 | -1.2, -1.1 |
| Never cancer: age, centered at 75 (quadratic) | 0.0 | 0.0, 0.0 | 0.0 | 0.0, 0.0 | 0.0 | 0.0, 0.0 | 0.0 | 0.0, 0.0 |
| Cancer: indicator | 0.1 | -2.7, 3.0 | 1.8 | -1.4, 5.0 | 0.0 | -3.1, 3.2 | -3.9 | -8.5, 0.7 |
| Cancer: age at cancer, centered at 75 | -0.5 | -0.7, -0.3 | -0.3 | -0.6, -0.1 | -1.0 | -1.2, -0.7 | -0.6 | -0.9, -0.2 |
| Cancer: years before cancer | -0.7 | -1.0, -0.4 | -0.4 | -0.8, -0.1 | -0.6 | -1.0, -0.3 | -0.8 | -1.2, -0.4 |
| Cancer: cancer now | -2.1 | -5.5, 1.2 | -12.8 | -18.9, -6.8 | -4.0 | -7.5, -0.5 | -5.4 | -10.7, -0.1 |
| Cancer: years after cancer | -0.9 | -1.3, -0.5 | -0.8 | -1.8, -0.2 | -1.2 | -1.7, -0.7 | -1.1 | -1.8, -0.5 |

**Note**: Values within ±0.01% are shown as 0.0% to maintain one decimal place.
